# Oxytocin increases physiological linkage during group therapy for methamphetamine use disorder: a randomized clinical trial

**DOI:** 10.1038/s41598-021-99957-8

**Published:** 2021-10-25

**Authors:** Katherine R. Thorson, Scott M. McKernan, Tessa V. West, Joshua D. Woolley, Wendy Berry Mendes, Christopher S. Stauffer

**Affiliations:** 1 Department of Psychology, Barnard College of Columbia University, 3009 Broadway, New York, NY 10027 USA; 2 Department of Psychiatry, University of California San Francisco, San Francisco, CA USA; 3 Department of Psychology, New York University, New York, NY USA; 4 Department of Psychiatry, Oregon Health & Science University, Portland, OR USA; 5 Portland VA Medical Center, 1601 E 4th Plain Blvd (V3SATP), Vancouver, WA 98661 USA; 6 San Francisco Veterans Affairs Health Care System, 4150 Clement St, San Francisco, CA 94121 USA

**Keywords:** Psychology, Social neuroscience, Randomized controlled trials

## Abstract

Patients and psychotherapists often exhibit behavioral, psychological, and physiological similarity. Here, we test whether oxytocin—a neuropeptide that can enhance expressivity and social perception—influences time-lagged “linkage” of autonomic nervous system responses among participants and facilitators during group therapy. Physiological linkage estimates (*n* = 949) were created from ten cohorts, each with two facilitators (*n* = 5) and four to six participants (*n* = 48), over six weekly sessions of group therapy for methamphetamine use disorder. All participants of a cohort received oxytocin or placebo intranasally in a randomized double-blind procedure before each session. Cardiac interbeat intervals (IBI) were measured continuously during sessions to estimate physiological linkage, operationalized as one cohort-mate’s IBI reactivity during one minute predicting another cohort-mate’s IBI reactivity during the following minute. In oxytocin cohorts, participants and facilitators experienced significant physiological linkage to their cohort-mates (i.e., their physiological responses were predicted by the prior responses of their cohort-mates) and significantly more linkage than people in placebo cohorts. Both effects occurred during the first and second sessions but not later sessions. Results suggest that oxytocin may enhance psychosocial processes often associated with linkage—such as social engagement—in groups and highlight oxytocin’s potential to improve group cohesion during group therapy.

Clinical Trials Registration: NCT02881177, First published on 26/08/2016.

## Introduction

Patients and psychotherapists often exhibit similarity across behavioral, psychological, and physiological responses, which can be important for developing therapeutic alliance and helping patients learn skills modeled by their therapists^1,2^. Synchrony of autonomic nervous system (ANS) responses has been of particular interest to researchers given its ability to develop quickly in new relationships and to reveal interpersonal dynamics that cannot otherwise be easily measured or even consciously known by patients and therapists^3–8^. Early work focused on how similarity between patients’ and therapists’ heart rates mapped onto behavioral processes such as rapport and antagonism^9,10^. More recent work has used experimental approaches to illuminate the processes that facilitate synchrony between therapists and patients^11,12^. Because physiological synchrony has been tied to social processes that are important within psychotherapy—including engagement with others^11,13^, empathic accuracy^14,15^, interpersonal emotion regulation^16,17^, and relationship quality^18–21^—understanding the conditions that foster synchrony in a therapeutic setting could help reveal pathways for improving the process of therapy and its outcomes.

In the current research, we test whether the neuropeptide oxytocin influences physiological synchrony among group members during group psychotherapy. Specifically, we examine moment-to-moment physiological “linkage” of autonomic nervous system (ANS) responses in ten cohorts undergoing treatment for methamphetamine use disorder (MUD) using motivational interviewing group therapy (MIGT). Methamphetamine is a highly addictive, potent psychostimulant, and the treatment options for methamphetamine users are often underutilized. We used MIGT because the primary barriers to accessing^22^ and maintaining^23^ treatment for methamphetamine use disorder are psychosocial in nature. Given that MIGT is a therapeutic modality designed to create a supportive client-centered social context for people with substance use disorders^24^, it seemed particularly likely that this type of therapy would help people remain in treatment for MUD. We tested the influence of oxytocin alongside MIGT in this clinical trial for two reasons. One, a common symptom of methamphetamine abuse and early abstinence is impaired social perception; for example, methamphetamine users struggle with accurately detecting emotions in others and inferring the intentions of others^25–28^. Given oxytocin’s putative ability to heighten the salience of social signals^29–33^, we reasoned that oxytocin might effectively address this symptom and improve social perception for methamphetamine users. Two, although we do not investigate these outcomes in this paper, oxytocin has demonstrated anti-addiction properties within humans for several other substances^34^ and has been shown to attenuate seeking and self-administration of methamphetamine within animal models^35–41^. Thus, we reasoned that oxytocin may also show anti-addiction properties for methamphetamine use in humans as well.

In this trial, each cohort met for six weekly therapy sessions and was composed of four to six participants and two co-facilitators. We measure physiological linkage as the extent to which the physiological response of each group member predicts the response of each other group member at a later time interval^5^ (described below). Although prior evidence has indicated that oxytocin enhances other forms of interpersonal synchrony within dyadic interactions^42–45^, to our knowledge, no prior work has tested the influence of oxytocin on physiological linkage of ANS responses during group psychotherapy.

There are several reasons to expect that oxytocin might enhance physiological linkage during group therapy sessions. First, researchers have theorized that interaction partners can become linked in their physiological responses when one partner (the “sender”) is expressive and provides affective information regarding their psychological and physiological experiences to the other partner (the “receiver”)^5,6,8^. This information about the sender, which could come through a variety of channels, such as behaviors, facial expressions, or speech, could cause the receiver to experience a similar physiological response. Given that oxytocin has been associated with increases in expressiveness of facial and vocal signals of emotion^46,47^, it may enhance physiological linkage between people by promoting greater expressiveness on the part of the sender.

Second, researchers have also theorized that physiological linkage can occur when people are perceptive of each other’s behaviors and social cues. Supporting this perspective, recent work has shown that linkage is associated with social processes indicative of being perceptive of others, including empathic accuracy^14^. Given that oxytocin can heighten the salience of social signals and improve social perception^29–33^, it may, therefore, also enhance physiological linkage between people by promoting better social perception. Importantly, oxytocin does not always improve social perception, and the strength and direction of oxytocin’s effects on social perception can vary widely depending on individual characteristics and social context^30,33,48–51^. Specifically, the positive effects of oxytocin on social perception tend to be limited to positive, affiliative social signals^52–55^ and to occur most strongly among people experiencing deficits in social cognition or perception^30,56–60^. Because both of these conditions were met in this study—MIGT aims to create a supportive client-centered social context^43^, and methamphetamine users often experience impairments in social perception^25,27,28,61^—we reasoned that oxytocin would be particularly likely to enhance physiological linkage in the context of this study.

## Research overview

We conducted a randomized, double-blind, placebo-controlled trial of oxytocin-enhanced MIGT^62,63^. Therapy participants were 48 men who have sex with men (MSM) and all met criteria for severe methamphetamine use disorder. They were assigned to a group cohort with four to six other participants and two co-facilitators. Participants were randomized to receive intranasal oxytocin or intranasal placebo before each weekly group therapy session over the course of six weeks. Each participant of a given cohort received the same study drug as the other participants in that cohort, and each cohort received the same study drug for all six sessions. Facilitators were not administered the study drug. Throughout the sessions, ANS responses were continuously collected from participants and facilitators. Prior analysis of data from this study suggested that oxytocin did indeed influence psychosocial outcomes in this context. Specifically, participants in oxytocin cohorts reported less empathic failure and group conflict than those in placebo cohorts and were significantly more likely to attend group therapy sessions^63^.

### Measure of physiological linkage

**Figure 1:**
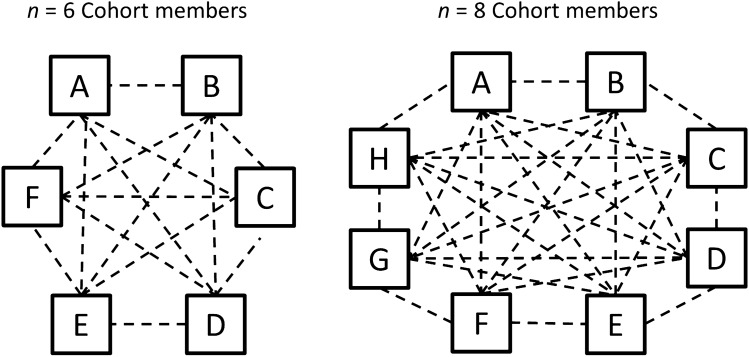
Dashed lines represent unique dyadic combinations in a cohort of six members (the minimum cohort size) and in a cohort of eight members (the maximum cohort size) during one session. Each dyadic combination has two linkage scores.

**Figure 2:**
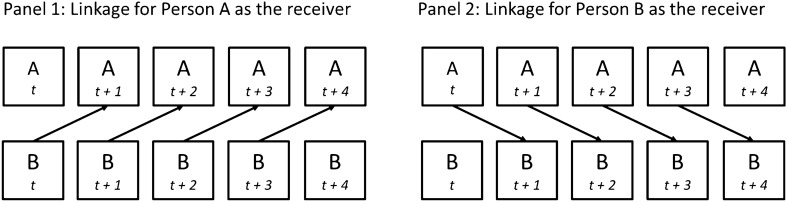
Physiological linkage for a dyad of Persons A and B over 5 min. (**Panel 1**) shows that Person A (as the “receiver”) has a linkage score that represents how much Person A’s physiological response at minute *t* + 1 is predicted by the physiological response of Person B (as the “sender”) at minute *t*, on average, throughout a group session. (**Panel 2**) shows that Person B also has a linkage score with Person A that represents how much Person B’s response at minute *t* + 1 is predicted by Person A’s response at minute *t*.

We calculated physiological linkage scores for each person in each dyadic combination per session per cohort using people’s cardiac interbeat interval reactivity from baseline as the physiological response of interest (described in more detail below; see Fig. 1). These linkage scores represent how strongly one person’s physiological responses were predicted by another group member’s responses from the prior minute, overall, within each session. For each dyadic combination, there can be two linkage scores (see Fig. 2), and all members of a cohort are both senders and receivers of physiological linkage, in all possible dyadic combinations.

We use this measure of physiological correspondence between people for three primary reasons. One, it utilizes a time-lagged component where one partner’s physiological response predicts another’s response at a following time interval, which we have theorized best captures how much people experience physiological changes as a result of their partners’ behaviors and social cues (in contrast to covariation models which examine simultaneous physiological responses^5^). Thus, this measure of synchrony best captures the processes through which we think oxytocin might affect linkage. Two, this measure allows us to examine how much certain factors—like the drug people received and their role in the therapy context (participant or facilitator)—affect linkage, whereas models that estimate one measure of similarity for two partners cannot do this. And, three, this approach allows us to adjust for physiological stability (i.e., how much people’s physiological responses are predicted by their own prior responses), which is important because stability accounts for a large share of the variance in people’s physiological responses at any given time interval^5,6,15,64^.

We assessed physiological linkage using cardiac interbeat intervals (IBI), which is the amount of time in milliseconds between successive heartbeats. Interbeat intervals are sensitive to quick changes in acute stress, motivation, and emotion (i.e., affective states), which we were interested in capturing within group members over time. They can also be obtained from multiple group members simultaneously and measuring them does not require people to inhibit their speech or movements. Because IBI represents a measure of general autonomic arousal and the intensity of people’s experiences, we interpret linkage on IBI responses as indicating the extent to which individuals “track” changes in the *intensity* of their partners’ psychological states through verbal and nonverbal cues their partners provide^65^. Although methamphetamine use is associated with differences in levels of ANS activity—specifically, greater sympathetic nervous system activity and less parasympathetic nervous system activity relative to healthy controls^63,66,67^—we adjusted for any of these potential differences by examining linkage of people’s interbeat interval reactivity from a baseline period. Thus, rather than examine the relationship between people’s raw or resting physiological levels (which can be different as a result of methamphetamine use), we instead examine the relationship between the physiological changes (i.e., reactivity) that people experience during group therapy relative to baseline.

### Hypotheses

#### Physiological linkage for participants

**Figure 3:**
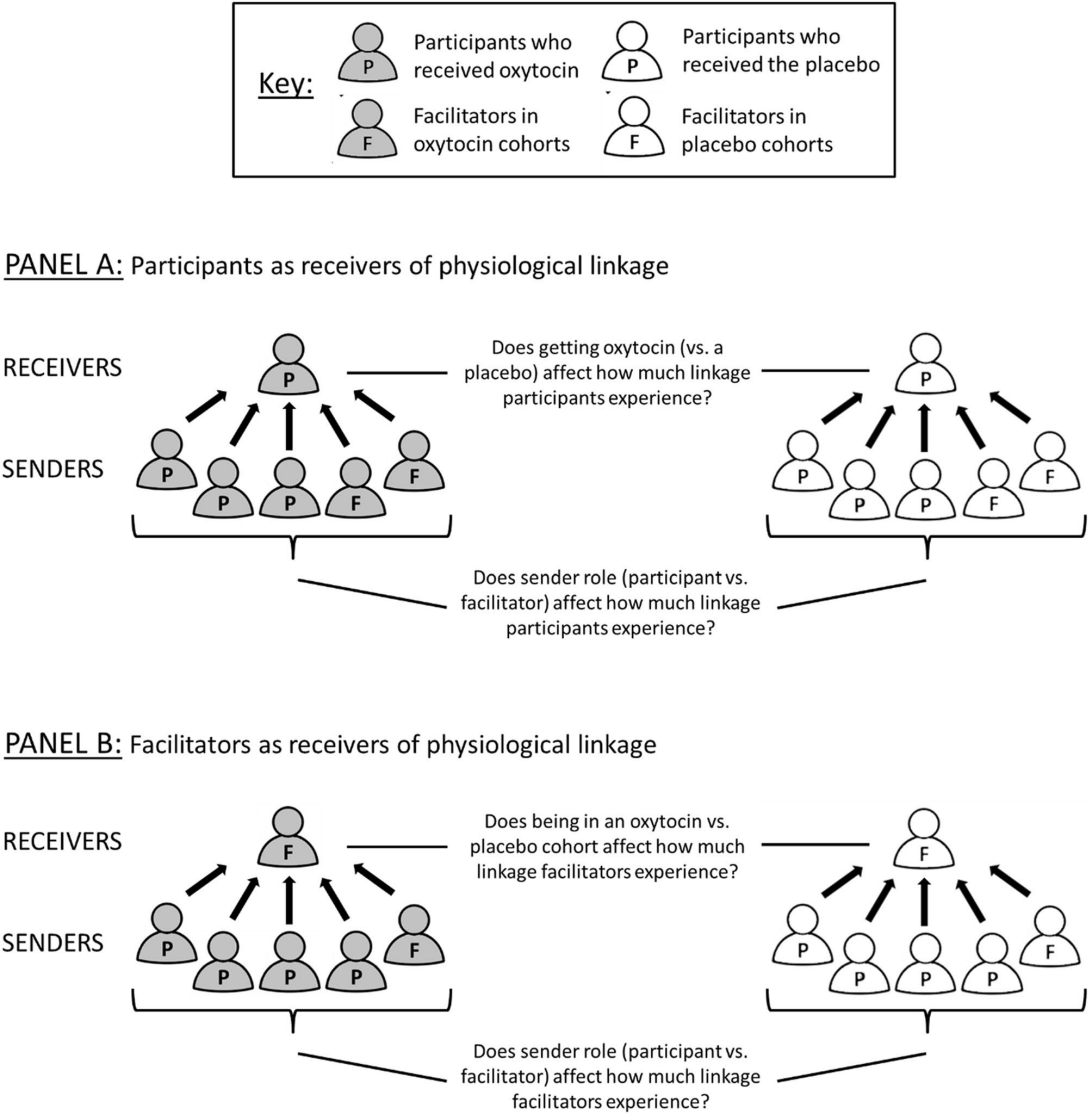
Models of physiological linkage. Arrows indicate potential physiological linkage from senders to receivers: senders’ physiological responses predict the physiological responses of receivers at a following time interval. (**A**) We examine how much participants are receivers of physiological linkage (i.e., how much other people’s physiological responses predict participants’ physiological responses) based on the drug they received and the role of senders. (**B**) We examine how much facilitators are receivers of physiological linkage (i.e., how much other people’s physiological responses predict facilitators’ physiological responses) based on the drug that participants in their cohort received and the role of senders.

Given oxytocin’s putative ability to enhance sensitivity to social cues, we predicted that participants who received oxytocin would experience significant physiological linkage to their cohort members. That is, we predicted that participants who received oxytocin would be “receivers” of physiological linkage and that their physiological responses would be significantly predicted by the prior responses of their cohort members (see Panel A of Fig. 3). We also predicted that oxytocin participants would experience more physiological linkage than participants who received a placebo. We explored whether these effects varied based on the role (participant vs. facilitator) of their other cohort members as “senders.” In other words, we explored whether the physiological responses of both participants and facilitators predicted the responses of participants who received oxytocin. On the one hand, other participants who were given oxytocin might be the only senders of linkage (if oxytocin only affects linkage by facilitating expressiveness, for example). On the other hand, participants and facilitators might both be senders of linkage (if oxytocin affects linkage by facilitating receivers’ sensitivity to social cues from anyone, for example).

#### Physiological linkage for facilitators

We also explored whether facilitators who were in cohorts where the participants received oxytocin (referred to as “oxytocin cohorts”) experienced significant physiological linkage to their cohort members and whether they experienced more physiological linkage than facilitators in cohorts where the participants received a placebo (referred to as “placebo cohorts”; see Panel B of Fig. 3). We explored whether the linkage experienced by facilitators in oxytocin vs. placebo cohorts occurred regardless of the role (participant vs. facilitator) of their other cohort members as “senders”. On the one hand, it is possible that facilitators in oxytocin cohorts would experience significant linkage to participants in their cohort if oxytocin promotes linkage by enhancing expressiveness in those participants. It is also possible that facilitators in oxytocin cohorts would experience significant linkage to other facilitators if oxytocin, by prompting greater expressiveness in participants, also prompts greater expressiveness in facilitators^68,69^. On the other hand, it is also possible that facilitators in oxytocin cohorts would experience no physiological linkage (regardless of sender role) if the effects of oxytocin on linkage are limited only to participants’ increased social sensitivity.

#### Physiological linkage over time

Finally, we examined whether linkage changed over the course of six group therapy sessions and whether these changes were influenced by cohort drug condition or role. As described above, we predicted that participants receiving oxytocin would experience more physiological linkage than participants receiving placebo (and we explored whether this was also the case for facilitators in those cohorts), but we thought it possible that this difference by drug condition might change over time. Some research testing the effects of repeated intranasal doses of oxytocin over time has shown no changes in the effects of oxytocin (on PTSD symptoms, for example) over time^70^. However, other work showing that oxytocin initially has effects on social cognition and emotion^60^ combined with work showing that these benefits are not present weeks and months later in similar populations^71,72^ suggest that the influence of oxytocin on psychosocial processes may, instead, decline over time. We explored both of these possibilities in the current study.

## Method

The protocol^62^ and main outcomes^63^ of this clinical trial (Clinical Trials Registration: NCT02881177, first published on 26/08/2016) have been published previously. The study was approved by the institutional review board at the University of California, San Francisco. All methods were carried out in accordance with relevant guidelines and regulations. Additional analytic details and results are provided in an Online Supplement; data and syntax are available at https://osf.io/8fhma/.

### Participants

Eligible participants were men who have sex with men (MSM) between the ages of 18 and 65, who were determined to have severe methamphetamine use disorder, using the *Diagnostic and Statistical Manual of Mental Disorders*, fifth edition. Participants entering the study were required to be treatment-seeking and have used methamphetamine at least once in the past 30 days. On average, participants had used methamphetamine 40% of the past 30 days. Participants were recruited from community health centers in San Francisco, California. All participants provided informed written consent prior to the study. There were ten MIGT cohorts; five cohorts received oxytocin (*n* = 24 participants), and five cohorts received placebo (*n* = 24 participants). Each cohort had two facilitators (a licensed psychiatrist and a mental health trainee—either a clinical psychology Ph.D./Psy.D. candidate or a psychiatry resident in their final year of training), who were not previously known to study participants; there were five different facilitators total. The general purpose of this early phase clinical trial was to investigate preliminary outcomes and evaluate the feasibility of our intervention; therefore, we set the sample size of 50 participants primarily for practical and clinical reasons.

### Procedures

Participants were randomly assigned to the oxytocin or placebo study drug condition (see Table 1). In a double-blind procedure, each participant in a given cohort received the same study drug as the other participants in their cohort prior to each weekly MIGT session, and participants received the same study drug for all six sessions. Facilitators did not receive a study drug. We report the percentage of participants who attended each session in Table 2; facilitators attended all sessions. Sessions 1–5 were 90 min, and session 6 was 60 min to provide participants with additional time after the session to complete final questionnaires.

**Table 1:** Number of people per cohort.

Number of Cohorts	Total cohort size at session 1	Number of participants per cohort	Number of facilitators per cohort
4	6	4	2
4	7	5	2
2	8	6	2

**Table 2:** Percentage of participants per cohort attending each session.

Cohort	Session						Mean
	1	2	3	4	5	6	
**Oxytocin**							
1	100%	100%	100%	100%	100%	100%	100
2	100%	100%	67%	83%	83%	83%	86
3	100%	100%	100%	100%	75%	75%	92
4	100%	80%	60%	20%	20%	20%	50
5	100%	100%	80%	80%	60%	80%	83
**Placebo**							
1	100%	100%	40%	60%	40%	60%	67
2	100%	100%	75%	50%	75%	100%	83
3	100%	75%	75%	75%	75%	75%	79
4	100%	50%	67%	100%	33%	67%	69
5	100%	60%	80%	80%	80%	100%	83

### Motivational interviewing group therapy (MIGT)

We adapted a treatment manual from Wagner and Ingersoll^24^ for a six-session course of MIGT centering on methamphetamine harm reduction and maximizing the potential benefits of intranasal oxytocin^63^. Motivational interviewing is a collaborative, person-centered, conversation style that honors autonomy and partnership, positioning the participant as the expert of their own story and evoking their own motivation and commitment to change. Participants generate their own goals (e.g., reduce or discontinue methamphetamine use), along with personalized plans for attaining stated goals (e.g., reconnect with supportive social networks). Co-facilitators normalize the fact that participants in each cohort may be at different stages of change and have different goals and plans for treatment compared to other members of their cohort. Fidelity ratings were acceptable and reported previously^63^.

### Study drug

Study drug was oxytocin 40 International Units (IU) or matched placebo and was administered intranasally by the study physician 30 min prior to each MIGT session.

### Physiology recording

All participants and facilitators were equipped with a Zephyr BioHarness v3.0 (Zephyr Technology, Auckland, New Zealand) prior to each session. The Zephyr TEAM System OmniSense Software (Version 4.2.4; https://www.zephyranywhere.com/) recorded continuous electrocardiography data sampled at 250 Hz. Prior to study drug administration, all participants and co-facilitators sat quietly for a 5-min baseline recording. We processed all ECG data in one-minute intervals using MindWare heart rate variability software (Version 3.1.2, MindWare Technologies, Ltd., Gahanna, OH, USA; https://mindwaretech.com/). We chose one-minute intervals because they are long enough to capture changes in IBI due to psychological experiences and because they can capture linkage that occurs during the natural exchanges in conversations^8^. We identified artifacts within each segment via MindWare’s dual MAD/MED and IBI Min/Max artifact detection algorithms^73^. We manually reviewed all software-identified R peaks and artifacts and edited them for accuracy. Each one-minute segment required at least 30 contiguous seconds of readable data to be included in analysis. IBI data were marked as missing when they could not be processed due to noise or loss of signal and when a participant missed a session. A total of 44.1% of IBI observations were missing (49.4% in placebo cohorts and 38.7% in oxytocin cohorts; see the Supplement for a version of our primary analysis conducted with maximum likelihood estimation to account for missing data, which shows results consistent with those presented below). We computed reactivity scores by subtracting the mean IBI from the last 60-s segment of baseline from the mean IBI of each 60-s segment of the group sessions.

### Physiological linkage

We calculated physiological linkage estimates for each unique dyadic combination per session per cohort to examine how much one dyad member’s IBI reactivity scores were predicted by each of their cohort-mates’ IBI reactivity scores during each session. We also adjusted for stability—receivers’ own prior physiology—when calculating linkage, based on the stability and influence model^74^. We estimated a regression model for each person in each dyad in each session in each cohort (see Eq. (1) for person *i* in dyad *j* at session *k* in cohort *l*), predicting the receiver’s (person *i*’s) reactivity score at minute *t* + 1 (*Y*_*ijkl(t*+*1*)_) from the partner’s (the sender’s) reactivity score at minute *t* (*S*_*ijklt*_) and the receiver’s own reactivity score at minute *t* (*R*_*ijklt*_). The coefficient *b*_*1ijkl*_ represents the linkage score for person *i* in dyad *j* at session *k* in cohort *l*.

1 $$Y_{ijkl(t + 1)} = \, b_{0ijkl} + \, b_{1ijkl} \times S_{ijklt} + \, b_{2ijkl} \times R_{ijklt} + \, e_{ijkl(t + 1)} .$$

Linkage estimates were calculated for each session. We marked as missing any linkage estimates that were created from fewer than 50% of all possible observations for that session (6.3% of the linkage estimates created) and any linkage estimates that were more extreme than three standard deviations from the mean linkage estimate^56^. After doing this, we identified and marked as missing two additional outliers which were, at that point, eight standard deviations from the mean and more than 2.5 standard deviations from the next closest score. In total, we analyzed 949 linkage observations, each of which was created from a minimum of 45 (Sessions 1 through 5) or 30 (Session 6) IBI reactivity estimates from two people each.

### Statistical methods

**Figure 4:**
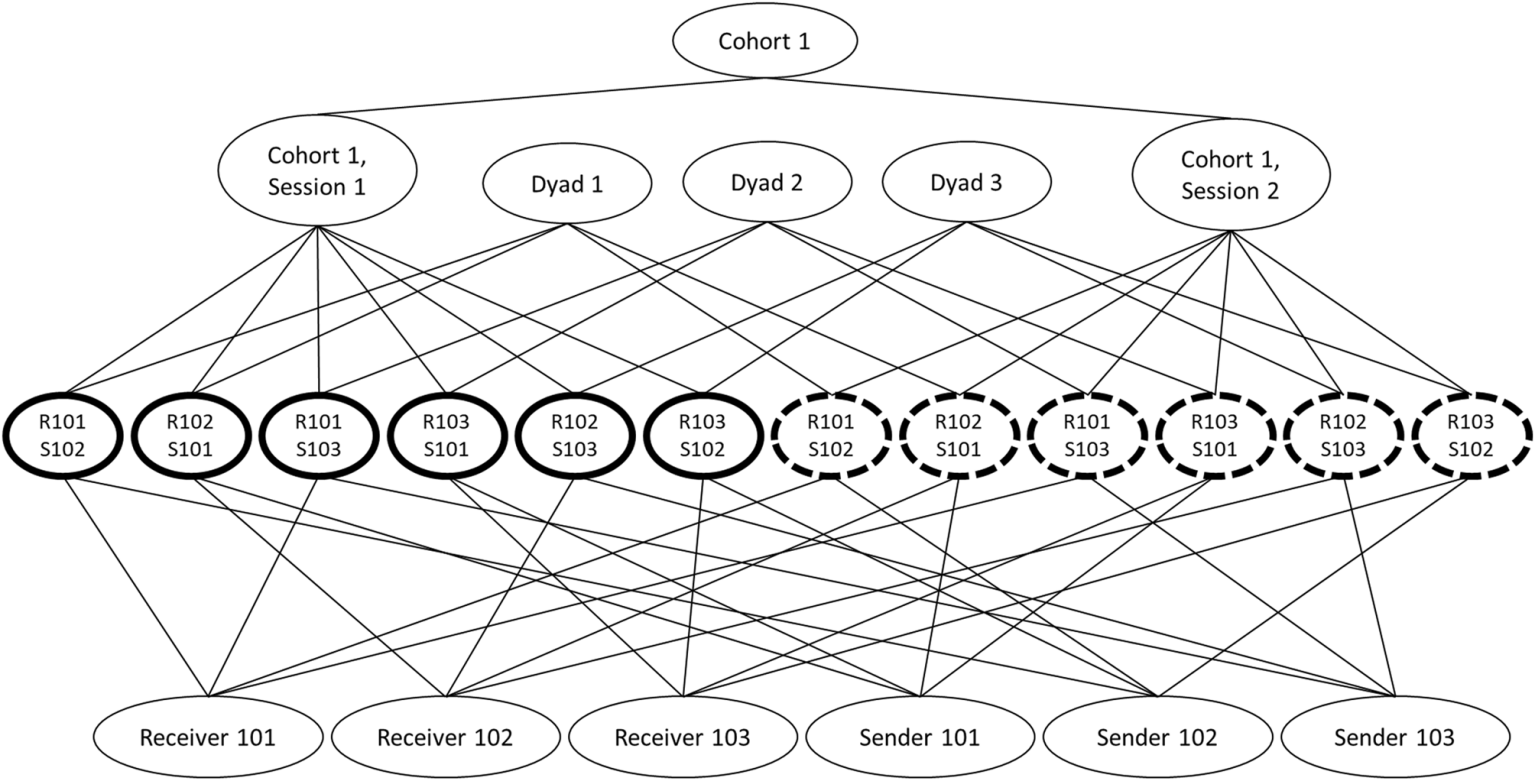
Structure of linkage estimates, displayed using an example of one cohort with two sessions and three people. Solid bold outlines indicate linkage estimates for Cohort 1 during Session 1; dashed bold outlines indicate linkage estimates for Cohort 1 during Session 2. The label of “R101, S102” indicates a linkage estimate for receiver 101 with sender 102. Dyads, receivers, and senders are not hierarchically nested within session because they are associated with linkage estimates across multiple sessions. Similarly, dyads, receivers, and senders are also not hierarchically nested within cohort because some facilitators participated in more than one cohort (see Supplement for more information).

We estimated a multilevel, cross-classified model^75,76^, adjusting for nonindependence in people’s linkage estimates due to cohort, session within cohort, dyad, receiver, and sender via random effects (see Fig. 4 for the structure of the data and the Supplement for a full list and description of the random effects included). To test whether physiological linkage varied as a function of cohort drug condition (a variable reflecting whether people were part of a cohort in which participants received oxytocin or placebo), receiver role, sender role, and session, we included these variables, and all possible interactions between them, as fixed effect predictors in the model. Session was a linear, continuous predictor. Given that all effects (other than those including session) refer to whatever session is currently coded as zero, we first report whether cohort drug condition, receiver role, and sender role are significant at the study midpoint (coded as zero) and whether they vary across sessions. If an effect varied across sessions, we then report the significance of that effect at each session. All tests are two-tailed, and we used an alpha of 0.05 to determine significance. We report effect sizes for fixed effects as partial-*R*^2^s, which are appropriate for multilevel models^77^. Three sensitivity analyses, which show that the effects reported below are consistent with a slightly different random effects structure, when extreme values are winsorized, and when maximum likelihood estimation is used to account for missing data, are reported in the Supplement.

## Results

**Figure 5:**
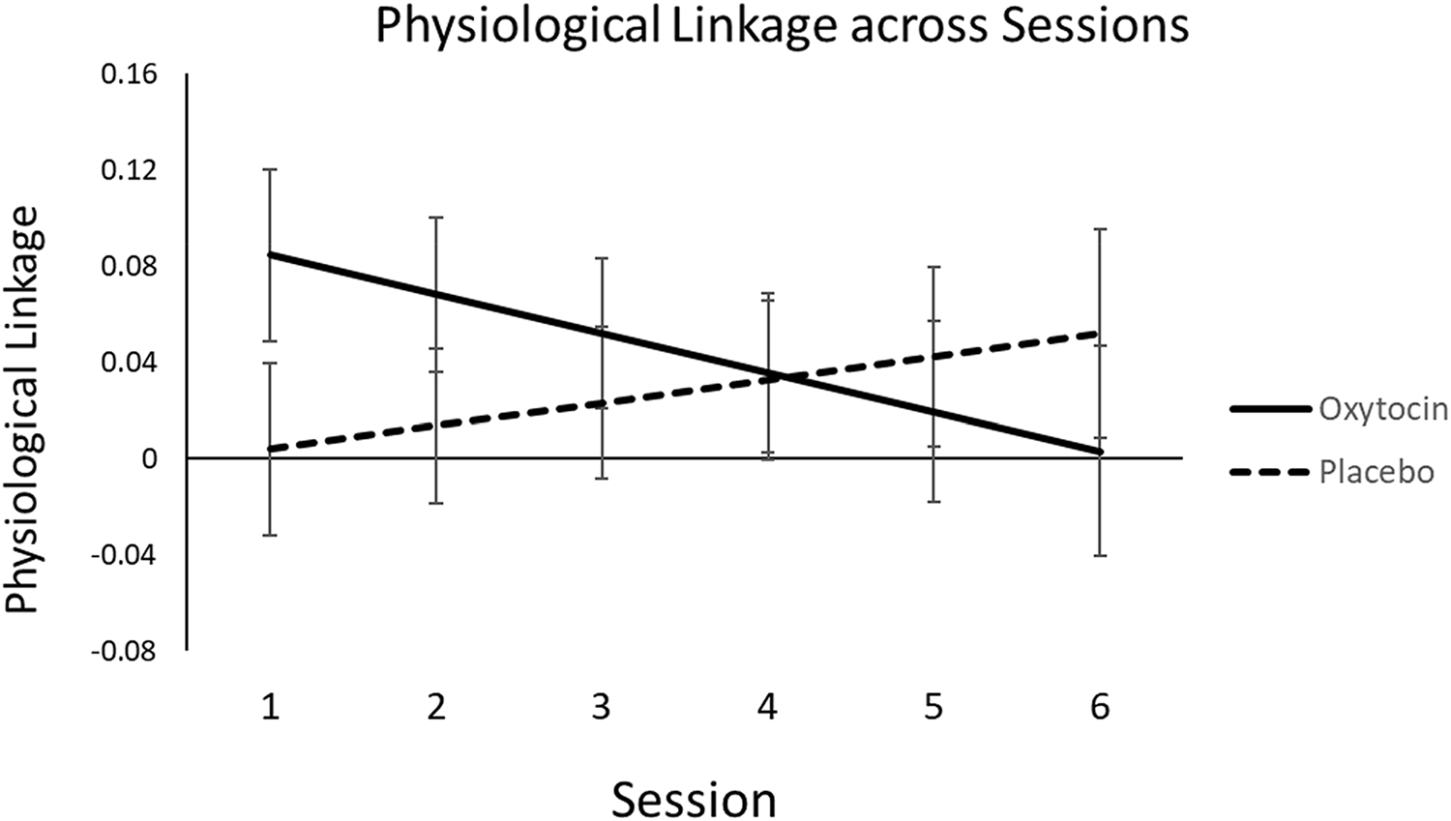
Physiological linkage by cohort drug condition across sessions. Bars represent standard errors.

**Figure 6:**
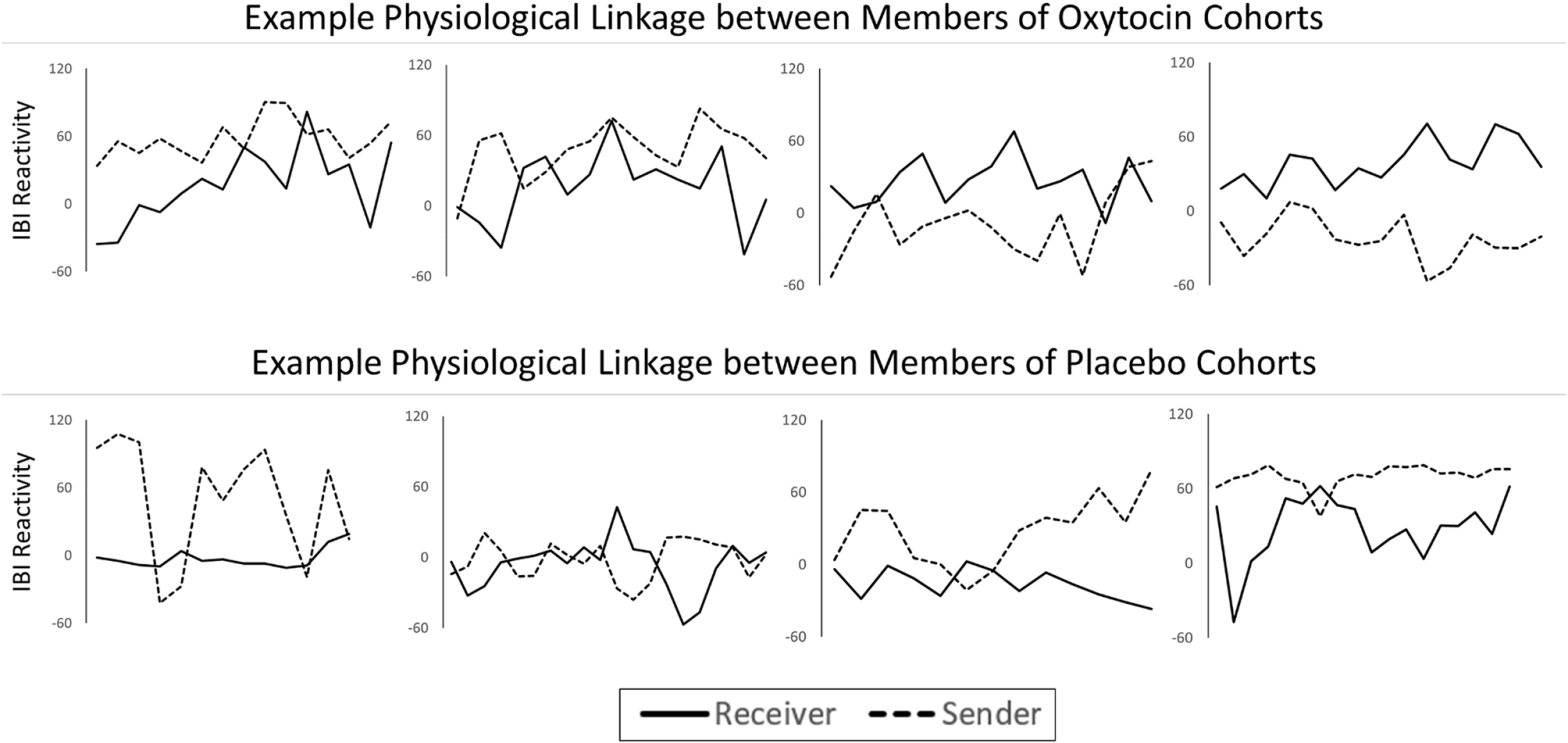
Example IBI reactivity patterns in oxytocin and placebo cohorts. Linkage occurs when the sender’s IBI reactivity predicts the receiver’s IBI reactivity at the next time interval.

First, we examined whether cohort drug condition influenced physiological linkage, on average, across both participants and facilitators. There was no main effect of cohort drug condition, *F*(1, 83.7) = 0.38, *p* = 0.54, ${R}_{\beta }^{2}$ = 0.005, but there was a significant interaction between cohort drug condition and session, *F*(1, 73.9) = 4.95, *p* = 0.029, ${R}_{\beta }^{2}$ = 0.06 (see Fig. 5). The linear change in linkage across sessions was not significant for oxytocin cohorts (*b* =  − 0.02, *SE* = 0.01, *t*(26) =  − 1.69, *p* = 0.10, 95% CI − 0.04 to 0.04, ${R}_{\beta }^{2}$ = 0.10) nor placebo cohorts (*b* = 0.01, *SE* = 0.01, *t*(25.6) = 1.02, *p* = 0.32, 95% CI − 0.01 to 0.03, ${R}_{\beta }^{2}$ = 0.04). However, during the first and second sessions, people in oxytocin cohorts showed significant linkage to their cohort-mates, *b* = 0.08, *SE* = 0.04, *t*(68.2) = 2.37, *p* = 0.021, 95% CI 0.01 to 0.16, ${R}_{\beta }^{2}$ = 0.08 (first session) and *b* = 0.07, *SE* = 0.03, *t*(50.6) = 2.13, *p* = 0.038, 95% CI 0.004 to 0.13, ${R}_{\beta }^{2}$ = 0.08 (second session), meaning that their physiological responses were significantly predicted by their cohort-mates’ responses at the prior time interval (see Fig. 6 for example IBI reactivity patterns). In contrast, people in placebo cohorts did not show significant linkage to their cohort-mates, *b* = 0.004, *SE* = 0.04, *t*(65.6) = 0.11, *p* = 0.91, ${R}_{\beta }^{2}$ = 0.0002 (first session) and *b* = 0.01, *SE* = 0.03, *t*(48.6) = 0.42, *p* = 0.68, 95% CI − 0.08 to 0.05, ${R}_{\beta }^{2}$ = 0.004 (second session). The difference in linkage between oxytocin and placebo cohorts was significant during the first session, *F*(1, 83.3) = 4.83, *p* = 0.031, ${R}_{\beta }^{2}$ = 0.05, and, although not statistically significant, was close to the cutoff for statistical significance during the second session *F*(1, 87.7) = 3.43, *p* = 0.067, ${R}_{\beta }^{2}$ = 0.04. At all future sessions, there was no influence of cohort drug condition on physiological linkage, *p*s > 0.24, and people in neither the oxytocin nor placebo cohorts showed linkage significantly greater than zero, *p*s > 0.10.

We did not find an influence of people’s role (participant vs. facilitator) on physiological linkage. Specifically, there was no main effect of receiver role (*F*(1, 13) = 2.01, *p* = 0.18, ${R}_{\beta }^{2}$ = 0.14), sender role (*F*(1, 21.8) = 0.66, *p* = 0.43, ${R}_{\beta }^{2}$ = 0.03), nor an interaction of the two, (*F*(1, 65.4) = 1.39, *p* = 0.24, ${R}_{\beta }^{2}$ = 0.05), and none of these effects varied significantly across sessions (receiver role by session: *F*(1, 233) = 0.14, *p* = 0.70, ${R}_{\beta }^{2}$ = 0.001; sender role by session: *F*(1, 9.37) = 0.10, *p* = 0.76, ${R}_{\beta }^{2}$ = 0.01; receiver role by sender role by session: *F*(1, 188) = 0.14, *p* = 0.71, ${R}_{\beta }^{2}$ = 0.01). Finally, none of the above role effects varied as a function of cohort drug condition (*p*s > 0.10).

Taken together, these results indicate that, during the first and second sessions, the physiological responses of both participants and facilitators in oxytocin cohorts were significantly predicted by the prior responses of their cohort-mates (and significantly more so than in placebo cohorts), and this did not vary by whether their cohort-mates were participants or facilitators. In other words, during the first and second sessions, when participants in a cohort received oxytocin, it facilitated (on average and relative to a placebo) physiological linkage for everyone in the cohort with every other member of their cohort.

## Discussion

We investigated physiological linkage during a six-week, double-blind, placebo-controlled trial of oxytocin-enhanced group therapy for people with methamphetamine use disorder. During group therapy sessions, we found that participants who received oxytocin experienced significant physiological linkage to their other cohort members—meaning that their physiological responses were significantly predicted by the prior responses of their other cohort members (both of other participants and facilitators). They also experienced significantly more physiological linkage than people in placebo cohorts. These two effects were evident during the first and second (out of six) therapy sessions but were not present during later sessions. Notably, these effects extended to group facilitators as well: in cohorts in which participants received oxytocin, facilitators also experienced more physiological linkage to their other cohort members (both participants and other facilitators) and more linkage than people in placebo cohorts. In sum, during the first and second therapy sessions, when participants in a given cohort received oxytocin, it facilitated (on average and relative to a placebo) physiological linkage for everyone in the cohort with every other member of their cohort.

Despite evidence that oxytocin can enhance other forms of interpersonal synchrony, to our knowledge, this is the first work to demonstrate that oxytocin can contribute to increased synchrony of people’s physiological responses during group therapy. To the extent that physiological synchrony in this context reflects greater attentiveness to and attunement with other people’s psychological experiences, these results suggest that administering oxytocin alongside group therapy may have beneficial effects on outcomes such as therapeutic alliance and group cohesion^18,21,78^. Publication of our main results demonstrated significant effects of oxytocin on session attendance and some measures of group cohesion^63^. Future work might consider the extent to which physiological linkage is associated with later outcomes and outcomes among other clinical populations as well.

Interestingly, although only participants received oxytocin in this study (facilitators were not administered any study drug), we found that the effects of oxytocin on physiological linkage extended to facilitators as well. In other words, even though facilitators did not receive oxytocin, when they were part of cohorts in which participants received oxytocin, they also experienced more physiological linkage in the first two sessions. This aligns with other research on oxytocin demonstrating that the effects of oxytocin administration in one interaction partner can affect psychosocial processes in the other partner as well^79,80^.

We see two potential reasons for the finding that facilitators in oxytocin cohorts experienced greater linkage than facilitators in placebo cohorts. One, participants who received oxytocin may have been more expressive regarding their psychological experiences^46,47^, which may have allowed facilitators to experience more physiological linkage with them (i.e., the responses of participants who received oxytocin may have been more likely to predict the responses of facilitators^5^). In addition, greater expressiveness on the part of participants may have resulted in greater expressiveness from facilitators as well, given that there is often a high degree of reciprocity in expressive behaviors between interaction partners^68,69^. This potentially would have allowed facilitators to experience more physiological linkage with the other facilitator as well. Two, participants who received oxytocin may have been more attentive to all other cohort members, which might have resulted in behavioral processes—such as crosstalk among participants—that instigated greater attentiveness on the part of facilitators, as well. By paying more attention to all cohort members, facilitators might have experienced more physiological linkage with them. Indeed, both of these possibilities may have occurred, and future work might address how the processes of expressivity and attentiveness contribute to physiological linkage experienced by both participants and facilitators.

One question our data raise is why oxytocin only affected physiological linkage during the first and second therapy sessions. It is possible that participants habituated to the effects of oxytocin over time, causing it to have a less potent influence on some of the psychosocial processes that contribute to linkage over time. In addition, not all participants attended all of the group therapy sessions, and it is possible that attrition of some group members affected group dynamics in ways that attenuated physiological linkage (for example, by reducing feelings of group cohesion^78^). One question that future research might examine is whether the initial effects of oxytocin instigate other processes which lead to later outcomes of interest, even if the original effects of oxytocin do not remain. For instance, if oxytocin leads people to be more expressive regarding their psychological experiences (which might be contributing to physiological linkage in early sessions), this could have a host of downstream effects—for example, expressers may feel more understood and their other cohort members may show them greater empathic responding^81,82^—even if the initial levels of expressiveness do not remain.

We examined the effects of oxytocin on physiological linkage within a particular context—MIGT—and with a specific sample—men who have sex with men and who met criteria for severe methamphetamine use disorder. Past work has made it clear that the consequences of oxytocin and the antecedents of physiological linkage are both quite context- and person-specific^4,7,14,29,30,83^. In particular, we speculated that oxytocin might have been particularly effective given its tendency to have positive psychosocial effects for people experiencing impairments in social perception to begin with, as is often the case for those diagnosed with methamphetamine use disorder. Future work should address the extent to which the effects we observed here are applicable to other contexts and settings. For example, it is possible that oxytocin would be less effective in promoting physiological linkage during group therapy with relatively healthy individuals.

In conclusion, the current study demonstrates that intranasal administration of oxytocin to participants with severe methamphetamine use disorder can enhance synchrony of physiological responses in a time-lagged fashion among participants and facilitators during initial group therapy sessions. These results suggest that psychosocial processes often associated with linkage—such as engagement with and attentiveness to others—may be enhanced in group settings via oxytocin. Furthermore, they show the ability of oxytocin to affect not only the people to whom it is directly administered, but also the interaction partners of those people, through social interaction. Importantly, this work highlights the potential of oxytocin to improve group cohesion and other forms of interpersonal synchrony during group therapy for substance use disorders.

## Supplementary Information

Supplementary Information.

## Data Availability

Data and analysis syntax are available at https://osf.io/8fhma/.
