# Supplementary material for: Oxytocin increases physiological linkage during group therapy for methamphetamine use disorder: a randomized clinical trial
Source: Sci Rep. 2021 Oct 25;11:21004. doi: 10.1038/s41598-021-99957-8 (PMC8546069; doi:10.1038/s41598-021-99957-8)
Supplement: Supplementary file 1 — Supplementary Information. [file 41598_2021_99957_MOESM1_ESM.docx]

Oxytocin Increases Physiological Linkage during Group Therapy

for Methamphetamine Use Disorder: A Randomized Clinical Trial

Katherine R. Thorson^1,*^

Scott M. McKernan^2^

Tessa V. West^3^

Joshua D. Woolley^2^

Wendy Berry Mendes^2^

Christopher S. Stauffer^2,4,*^

1. Department of Psychology, Barnard College of Columbia University, New York, NY, USA
2. Department of Psychiatry, University of California San Francisco, San Francisco, CA, USA
3. Department of Psychology, New York University, New York, NY, USA
4. Department of Psychiatry, Oregon Health & Science University & Portland VA Medical Center, Portland, OR, USA

**Corresponding authors*

Christopher S. Stauffer, MD Katherine Thorson, PhD

1601 E 4^th^ Plain Blvd (V3SATP) 3009 Broadway

Vancouver, WA 98661 New York, NY 10027

(360) 696-4061 x32017 212-854-8272

Christopher.Stauffer@va.gov kthorson@barnard.edu

**Supplement**

**Physiological Linkage Analyses**

**Random effects estimated.** In addition to examining the influence of drug condition and role on physiological linkage over time, we also examined how much of the additional variance in linkage estimates was due to individual-level, dyad-level, and cohort-level effects (see the Social Relations Model; Kashy and Kenny, 2000). The random effects that we estimated in our final physiological linkage model are listed in Table S1. We were not able to estimate the following parameters: a random intercept for cohort, a random session slope for cohort, a random session slope for receiver, the covariance between the intercept and session slope for dyad, the covariance between the intercept and session slope for sender, nor any other interactions between random effects terms.

**Table S1.** Random Effects Estimated.

| Effect | Source | Estimate | *SE* | *Z* | *p* | Question addressed |
| --- | --- | --- | --- | --- | --- | --- |
| Intercept | Cohort session | 0.0007 | 0.0007 | 0.96 | .17 | Does the unique combination of a particular session (1 through 6) for a particular cohort (1 through 10) account for variability in linkage estimates? |
| Intercept | Dyad | 0.0003 | 0.0009 | 0.27 | .39 | Does the unique dyadic combination of a particular receiver with a particular sender account for variability in linkage estimates? |
| Slope for session | Dyad | 0.0001 | 0.0001 | 1.01 | .16 | Does the unique dyadic combination of a particular receiver with a particular sender account for variability in how linkage estimates change across sessions? |
| Intercept | Receiver | 0.0004 | 0.0004 | 0.92 | .18 | Do receivers account for variability in linkage estimates? |
| Intercept | Sender | 0.007 | 0.002 | 2.82 | .002 | Do senders account for variability in linkage estimates? |
| Slope for session | Sender | 0.0002 | 0.0002 | 1.01 | .16 | Do senders account for variability in how linkage estimates change across sessions? |
| Intercept | Cohort session by receiver | 0.003 | 0.001 | 2.45 | 0.007 | Does the amount of variance that receivers account for depend on which cohort session they are in? |
| Intercept | Cohort session by sender | 0.002 | 0.001 | 1.72 | .043 | Does the amount of variance that senders account for depend on which cohort session they are in? |
| Residual |  | 0.03 | 0.002 | 15.31 | < .0001 | Is there additional variance left to be accounted for? |

**Random effects results.** We found that senders account for a significant amount of variance in linkage scores, τ = 0.007, *SE* = 0.002, *Z* = 2.82, *p* = .002, but this depends on which cohort session senders are in, τ = 0.002, *SE* = 0.001, *Z* = 1.72, *p* = .043. In other words, the physiological responses of some participants consistently predict the responses of their cohort-mates more than the responses of other participants do, but the extent to which this happens also depends on which cohort session participants are in. We also found that whether receivers account for a significant amount of variance in linkage scores depends on which cohort session receivers are in, τ = 0.003, *SE* = 0.001, *Z* = 2.45, *p* = .007. In other words, the extent to which the physiological responses of some participants are consistently predicted by the responses of their cohort-mates depends on which cohort session participants are in. In sum, physiological linkage estimates vary across participants, and who the receiver is, who the sender is, and what the current cohort context is (i.e., the cohort session) all contribute to that variation.

**Sensitivity analyses.**

***Alternative nesting structure.*** In our physiological linkage model presented in the main text, dyads, receivers, and senders are not hierarchically nested within cohort because some facilitators participated in more than one cohort. To test the robustness of our effects, we estimated a model in which dyads, receivers, and senders were hierarchically nested within cohort. Effects were largely consistent with those reported in the main text and are listed below.

There was no main effect of drug condition, *F*(1, 66.9) = 0.33, *p* = .57, but there was a significant interaction between drug condition and session, F*(*1, 61.7) = 4.23, *p* = .044. The linear changes in linkage across sessions were not significant in the oxytocin (*t*(68.5) = -1.98, *p* = .052) nor placebo (*t*(55.7) = 0.92, *p* = 0.36) conditions. However, during the first, second, and third sessions, people in oxytocin cohorts showed significant linkage to their cohort-mates, *t*(101) = 2.87, *p* = .005 (first session), *t*(94.4) = 2.77, *p* = .007 (second session), *t*(76.6) = 2.36, *p* = .02 (third session), meaning that their physiological responses were significantly predicted by their cohort-mates’ responses at the prior time interval. Linkage was not significant at later sessions, *p*s > .11.

In contrast, people in placebo cohorts did not show significant linkage to their cohort-mates during any sessions, *p*s > .16. The difference in linkage between people in oxytocin versus placebo cohorts was close to the cutoff for statistical significance during the first session (*t*(98.3) = 1.91, *p* = .059. At all future sessions, there was no influence of drug condition on physiological linkage, *p*s > .12.

We did not find an influence of people’s role (participant vs. facilitator) on physiological linkage. There was no main effect of receiver role (*F*(1, 50.3 = 1.71, *p* = .20), sender role (*F*(1, 52.1) = 2.10, *p* = .16), nor an interaction of the two, (*F*(1, 92.6) = 1.27, *p* = .26), and none of these effects varied significantly across sessions (receiver role by session: *F*(1, 245) = 0.10, *p* = .76; sender role by session: *F*(1, 145) = 0.16, *p* = .69; receiver role by sender role by session: *F*(1, 204) = 0.13, *p* = .72). Finally, none of the above role effects varied as a function of drug condition (*p*s > .13).

***Winsorizing linkage estimates three standard deviations beyond the mean.*** To be certain that the effects reported in the main text were not driven by extreme values, we conducted a sensitivity analysis in which we winsorized all linkage estimates that were more than three standard deviations beyond the mean linkage estimate (within drug and within session). To winsorize, we replaced all values that were beyond three standard deviations from the mean with the value at three standard deviations beyond the mean (Table S2 shows the percentage of estimates that were winsorized within drug and within session).

**Table S2**

***Percentage of estimates three or more standard deviations from the mean***

| Session | Percentage of estimates three or more standard deviations below the mean | |  | Percentage of estimates three or more standard deviations above the mean | |
| --- | --- | --- | --- | --- | --- |
|  | Oxytocin | Placebo |  | Oxytocin | Placebo |
| Session 1 | 0.0% | 1.67% |  | 2.9% | 0.8% |
| Session 2 | 0.9% | 1.8% |  | 0.9% | 0.0% |
| Session 3 | 0.0% | 2.1% |  | 2.8% | 0.0% |
| Session 4 | 1.1% | 2.3% |  | 1.1% | 0.0% |
| Session 5 | 0.0% | 1.9% |  | 1.6% | 0.0% |
| Session 6 | 0.0% | 2.2% |  | 1.1% | 3.3% |

With the winsorized data, we followed the same analytic approach as in the main text, and all results were consistent with those reported in the main text. There was no main effect of drug condition, *F*(1, 94.3) = 0.34, *p* = .56, but there was a significant interaction between drug condition and session, *F*(1, 76.3) = 5.12, *p* = .027. The linear changes in linkage across sessions were not significant in the oxytocin (*t*(28.8) = -1.76, *p* = .09) nor placebo (*t*(28.4) = 1.02, *p* = 0.32) conditions. However, during the first and second sessions, people in oxytocin cohorts showed significant linkage to their cohort-mates, *t*(70.7) = 2.51, *p* = .014 (first session) and *t*(52) = 2.27, *p* = .028 (second session), meaning that their physiological responses were significantly predicted by their cohort-mates’ responses at the prior time interval. During the third session, people who received oxytocin showed linkage that, although not statistically significant, was close to the cutoff for statistical significance, *t*(38.8) = 1.77, *p* = .085.

In contrast, people in placebo cohorts did not show significant linkage to their cohort-mates, *t*(67) = 0.14, *p* = .89 (first session) and *t*(49.4) = 0.47, *p* = .64 (second session). The difference in linkage between oxytocin and placebo cohorts was significant during the first session, *F*(1, 85.5) = 5.02, *p* = .028, and marginally significant during the second session *F*(1, 97.3) = 3.57, *p* = .062. At all future sessions (sessions 4 through 6 for people in oxytocin cohorts and sessions 3 through 6 for people in placebo cohorts), there was no influence of drug condition on physiological linkage, *p*s > .22, and participants in neither the oxytocin nor placebo cohorts showed linkage significantly greater than zero, *p*s > .22.

We did not find an influence of people’s role (participant vs. facilitator) on physiological linkage. There was no main effect of receiver role (*F*(1, 13.2) = 2.42, *p* = .14), sender role (*F*(1, 22.3) = 0.71, *p* = .41), nor an interaction of the two, (*F*(1, 98.3) = 1.60, *p* = .21), and none of these effects varied significantly across sessions (receiver role by session: *F*(1, 259) = 0.14, *p* = .71; sender role by session: *F*(1, 10.3) = 0.13, *p* = .73; receiver role by sender role by session: *F*(1, 213) = 0.15, *p* = .71). Finally, none of the above role effects varied as a function of drug condition (*p*s > .10).

***Missing data.*** To account for missing data, we also conducted a version of our primary model using maximum likelihood (ML) estimation, which is a common method for providing parameter estimates that account for missing data. (This is in contrast to restricted maximum likelihood estimation, which is the estimation method we used in the main text because it is better at providing unbiased estimates of covariance parameters specifically; [Raudenbush & Bryk, 2002].) All results using ML estimation in the primary model were consistent with those

presented in the main text and are listed below.

There was no main effect of drug condition, *F*(1, 99.3) = 0.42, *p* = .52, but there was a significant interaction between drug condition and session, *F*(1, 79.9) = 5.49, *p* = .022. The linear changes in linkage across sessions were not significant in the oxytocin (*t*(18.6) = -1.88, *p* = .08) nor placebo (*t*(19.1) = 1.06, *p* = 0.30) conditions. However, during the first and second sessions, people in oxytocin cohorts showed significant linkage to their cohort-mates, *t*(76.2) = 2.51, *p* = .014 (first session) and *t*(55.8) = 2.27, *p* = .027 (second session), meaning that their physiological responses were significantly predicted by their cohort-mates’ responses at the prior time interval. During the third session, people in oxytocin cohorts showed linkage that, although not statistically significant, was close to the cutoff for statistical significance, *t*(38.6) = 1.80, *p* = .079.

In contrast, people in placebo cohorts did not show significant linkage to their cohort-mates, *t*(72.5) = 0.12, *p* = .90 (first session) and *t*(52.9) = 0.44, *p* = .66 (second session). The difference in linkage between people in oxytocin and placebo cohorts was significant during the first session, *F*(1, 89.7) = 2.28, *p* = .025, and marginally significant during the second session *F*(1, 93) = 1.95, *p* = .054. At all future sessions (sessions 4 through 6 for people in oxytocin cohorts and sessions 3 through 6 for people in placebo cohorts), there was no influence of drug condition on physiological linkage, *p*s > .23, and participants in neither the oxytocin nor placebo conditions showed linkage significantly greater than zero, *p*s > .21.

We did not find an influence of people’s role (participant vs. facilitator) on physiological linkage. There was no main effect of receiver role (*F*(1, 14.8) = 2.63, *p* = .13), sender role (*F*(1, 21.8) = 0.81, *p* = .38), nor an interaction of the two, (*F*(1, 104) = 1.68, *p* = .20), and none of these effects varied significantly across sessions (receiver role by session: *F*(1, 267) = 0.13, *p* = .71; sender role by session: *F*(1, 6.82) = 0.16, *p* = .70; receiver role by sender role by session: *F*(1, 229) = 0.16, *p* = .70). Finally, none of the above role effects varied as a function of drug condition (*p*s > .09).

**IBI Reactivity Analyses**

**Fixed effects estimated.** We estimated a multilevel model to test whether reactivity varied as a function of drug received by participants in the cohort (oxytocin vs. placebo), role (participant or facilitator), session, and time, we included these variables, and all possible interactions between them, as fixed effect predictors in the model. Session and time were both linear, continuous predictors but at different timescales. Session refers to the specific MIGT therapy session (1 through 6), and time refers to the minute within those individual sessions (1 through 90 for sessions 1 through 5, and 1 through 60 for session 6). We also included methamphetamine use as a covariate (to do this, participants provided a urine sample at each visit, which was screened for the presence of methamphetamine). We anticipated that all people would show decreases in reactivity over the course of individual therapy sessions, given expected habituation, so we first examined whether IBI reactivity varied over time. We then examined whether the effect of time varied as a function of drug, role, or session.

As expected, we found a significant effect of time, *b* = 0.27, *SE* = 0.06, *t*(8.22) = 4.36, *p* = .002, $R_{\beta}^{2}$ = 0.70, such that people’s IBI reactivity declined over the course of individual MIGT sessions (i.e., general ANS arousal decreased during individual MIGT sessions). We also found an effect of role that was close to the cutoff for statistical significance, *F*(1, 13.2) = 4.52, *p* = .053, $R_{\beta}^{2}$ = 0.26, such that facilitators (*M* = -21.93, *SD* = 71.87) showed stronger IBI reactivity than participants (*M* = 3.90, *SD* = 60.54). These effects were qualified by a significant time by role interaction: *F*(1, 195) = 15.78, *p* < .001, $R_{\beta}^{2}$ = 0.07, (see Figure S1). Participants’ reactivity declined over the course of individual sessions, *b* = 0.44, *SE* = 0.07, *t*(11) = 6.47, *p* < .001, 95% CI: 0.29 to 0.58, but facilitators’ IBI reactivity remained stable over the course of individual sessions, *b* = 0.11, *SE* = 0.08, *t*(22.4) = 1.37, *p* = .19, 95% CI: -0.06 to 0.28. The role by time interaction was not moderated by drug (*F*(1, 195) = 0.04, *p* = .84, $R_{\beta}^{2}$ = 0.0002), session (*F*(1, 216) = 2.14, *p* = .15, $R_{\beta}^{2}$ = 0.01), nor an interaction of the two (*F*(1, 216) = 1.49, *p* = .22, $R_{\beta}^{2}$ = 0.01). Aside from the significant effects of time and time by role, no other significant effects were found (*p*s > .11).


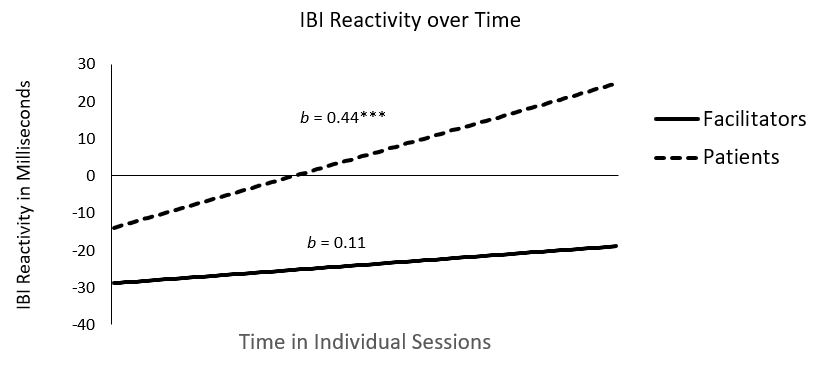


**Figure S1.** IBI reactivity over time during individual sessions.

**Random effects estimated.** The random effects that we estimated in our final reactivity model are listed in Table S3. We assumed that measurements adjacent in time would be correlated with each other, potentially due to factors unspecified in our model, so we specified an autoregressive structure for the Level 1 residuals for each person’s IBI reactivity over time within each session within each cohort to account for this (Bolger & Laurenceau, 2013). We were not able to estimate a random slope for session for each cohort, a random intercept for cohort session, random slopes for time or session for each person, covariances between any intercepts and slopes, or any additional random effect interactions.

**Table S3.** Random Effects Estimated in IBI Reactivity Model.

| Effect | Source | Estimate | *SE* | *Z* | *p* |  |
| --- | --- | --- | --- | --- | --- | --- |
| Intercept | Cohort | 73.28 | 102.65 | 0.71 | .24 | Does the cohort that people are in account for variability in IBI reactivity? |
| Slope for time | Cohort | 0.01 | 0.02 | 0.70 | .24 | Does the cohort that people are in account for variability in how IBI reactivity changes across sessions? |
| Slope for time | Cohort session | 0.04 | 0.03 | 1.63 | .052 | Does the unique combination of a particular session (1 through 6) for a particular cohort (1 through 10) account for variability in how IBI reactivity changes across sessions? |
| Intercept | Person | 283.81 | 191.43 | 1.48 | .070 | Do people account for variability in IBI reactivity (i.e., do some people show stronger IBI reactivity than others)? |
| Intercept | Cohort session by person | 2239.38 | 234.61 | 9.55 | < .001 | Does the amount of variance that people account for depend on which cohort session they are in? |
| Slope for time | Cohort session by person | 0.25 | 0.04 | 7.07 | < .001 | Does the amount of variance that people account for in how IBI reactivity changes over time depend on which cohort session they are in? |
| Residual |  | 1510.79 | 20.13 | 75.06 | < .001 | Is there additional variance left to be accounted for? |

**Sensitivity analysis.** In our IBI reactivity model, people were not hierarchically nested within cohort because some facilitators participated in more than one cohort. As a sensitivity analysis, we estimated a model in which people were hierarchically nested within cohort, and the effects were largely consistent with those reported above. The effect of time remained significant and positive, and this effect was again moderated by role. The main effect of role was significant, *F*(1, 57.2) = 11.52, *p* = .001. No other effects were significant.
